# The postbiotic of hawthorn-probiotic ameliorating constipation caused by loperamide in elderly mice by regulating intestinal microecology

**DOI:** 10.3389/fnut.2023.1103463

**Published:** 2023-03-16

**Authors:** Yu Wei, Na Huang, Xinyu Ye, Meng Liu, Meilian Wei, Yali Huang

**Affiliations:** ^1^Basic Medical Science College, Guangzhou University of Chinese Medicine, Guangzhou, Guangdong, China; ^2^The Eighth School of Clinical Medicine (Foshan Hospital of TCM), Guangzhou University of Chinese Medicine, Guangzhou, Guangdong, China

**Keywords:** postbiotic, hawthorn, probiotic, constipation, AQP3, Enac-**γ**, inflammation, intestinal microenvironment

## Abstract

**Background:**

Constipation is common gastrointestinal disorder with high prevalence and recurrence, making people suffering. However, the treatment for constipation remains ineffectual. We aimed to the study the effects and mechanisms of postbiotic of hawthorn-probiotic on loperamide modeled old KM mice.

**Methods:**

Constipated mice were grouped and treated with 10% lactulose (Y), hawthorn group (S), probiotic group (F) and postbiotic of hawthorn-probiotic (FS). Fecal changes were observed. AQP3 and Enac-γ were measured by RT-qPCR and Western blotting, intestinal barrier by H&E and immunofluorescence staining, cell proliferation and apoptosis by CCK8 and flow cytometry. Gut microbiota was further determined by 16 s rRNA sequence of feces.

**Results:**

Postbiotic of hawthorn-probiotic improved intestinal movement and pathomorphology, elevated AQP3, Enac-γ and mucin-2 expression, accompanied by decreased serum TNF-α and cell apoptosis, but increased proliferation. Furthermore, it modified the gut microbiota of constipated mice, featured by upregulation of *Lactobacillaceae*.

**Conclusion:**

Postbiotic of hawthorn-probiotic relieved constipation by combined effects of regulating intestinal water and sodium metabolism, maintain intestinal barrier and gut microflora.

**Graphical Abstract:**
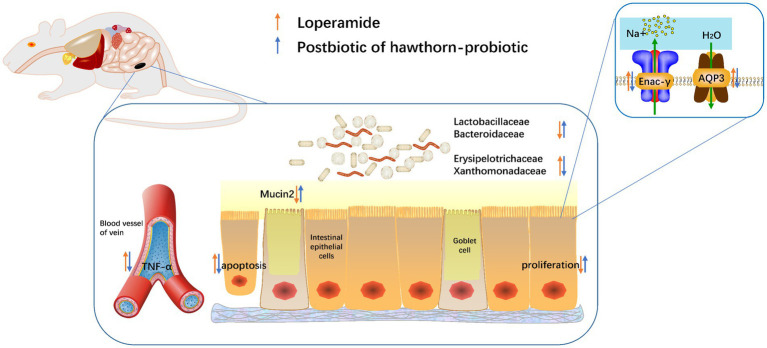

## 1. Introduction

Constipation is one of the most frequent gastrointestinal disorders. It’s reported that the global incidence of constipation has reached 15% just in 2020 (1), and the prevalence of constipation in the elderly is as high as 27% (2), and the prevalence increased with the increase of age (3). And this might be underestimated since partial patients do not seek medical help in hospitals, especially in developing countries. Meanwhile, constipation has a 50% recurrence rate (4). Constipation increases discomfort and may lead to abdominal cramping and straining on defecation that ultimately affect quality of life (5). Prolonged constipation can also lead to neurasthenia, metabolic disorders, and even sepsis (6). However, the treatment for constipation remains ineffectual. In recent years, studies have found that the intestinal microbial community of constipation patients has significantly changed compared with that of normal people (7, 8). It has also been found that supplementing with prebiotic (nutrients designed to stimulate the growth of beneficial microbes), probiotic (microbes that confer a health benefit when consumed at sufficient levels) or synbiotic (a combination of a prebiotic and a probiotic) can relieve constipation symptoms such as frequency of defecation, fecal characteristics, colonic mucosa, colonic transit, gut microbiota, metabolite (9–14). However, these rely on external supplements or the patient’s own live bacteria. Inanimate microorganisms and/or their components that are beneficial to host health are called postbiotic (15). It has been observed that the culture supernatants of certain probiotics maintain the same effectiveness of alive bacteria, furthermore, postbiotics are in some cases considered a valid and safer alternative to taking viable microorganisms (16).

Hawthorn is a kind of fruit, which has been widely used in the formulation of dietary supplements, functional foods and medical products. Hawthorn is rich in amino acids, minerals, pectin, vitamin C, polyphenols (chlorogenic acid, proanthocyanidin B2, epicatechin), flavonoids (proanthocyanidin, colloxanidine, quercetin, rutin) (17), because of which, hawthorn exerts extensive biological functions, like antioxidant, anti-inflammatory, anti-cancer, anti-cardiovascular disease and digestive promoting properties.

*Lactobacillu*s, one of the most beneficial probiotics in fermented food production, has been widely applied with a long-term history and safety (18). The most important function of *lactobacillu*s is to improve digestive and immune functions (19, 20). *Lactobacillus paracasei*, one of seven species in the genus *Lactobacillus*, has two subspecies (*L. paracasei* subsp. *paracasei* and *L. paracasei* subsp. *tolerans*) (21). It has been shown to be safe (22) and is used in the fermentation of dairy products and cheese (23, 24). And studies have found that using these live bacteria can relieve constipation (2, 25, 26). However, there is no report exploring the effects of its metabolites (postbiotic).

In this study, we aimed to explore the effects of postbiotic of hawthorn-probiotic on constipation. The *Lactobacillus paracasei* isolated from the feces of infants was cultured with aqueous extract of hawthorn. After fermentation, the postbiotic was obtained by removing the living bacteria. The effects and possible mechanisms of the postbiotic on constipated elderly mice induced by loperamide was then explored. We found that postbiotic of hawthorn-probiotic exerts remarkable effects on constipation by regulating water and sodium metabolism, repairing intestinal barrier, relieving inflammation, and restoring microflora structure. This might offer a promising therapy for constipation.

## 2. Results

### 2.1. Postbiotic of hawthorn-probiotic increased intestinal motivity

**Figure 1:**
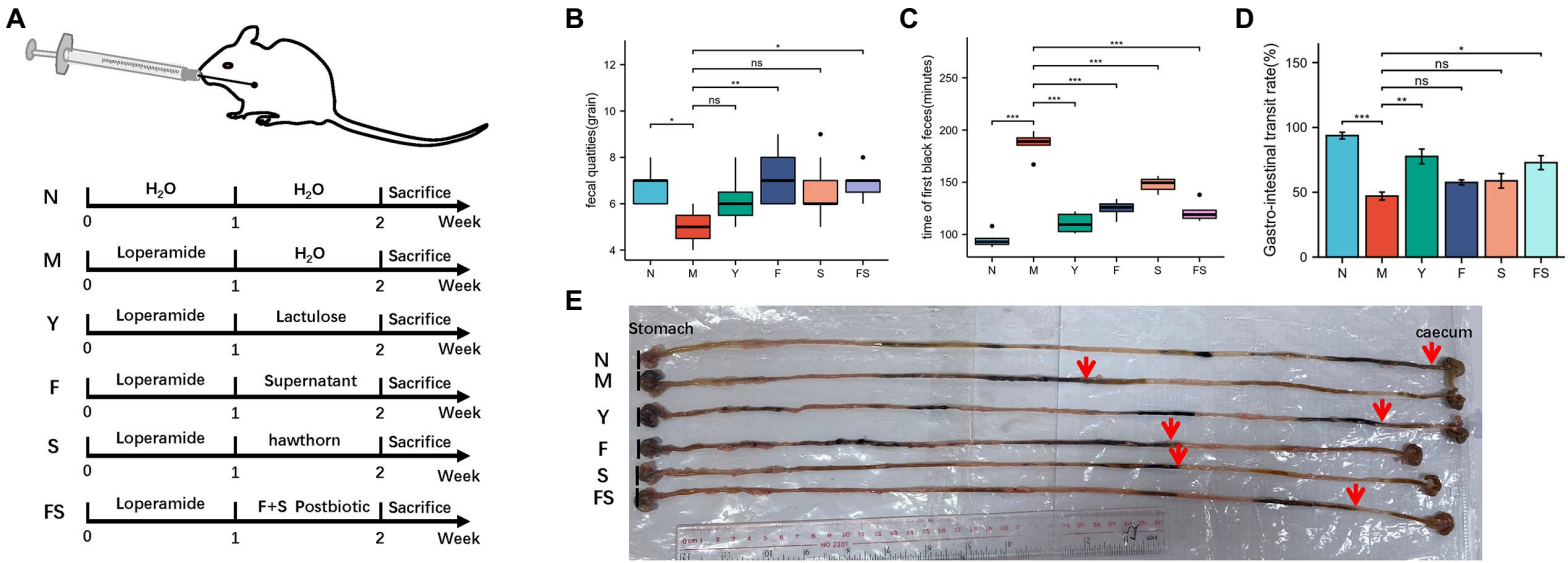
Postbiotic of hawthorn-probiotic can increase intestinal motivity. **(A)** Animal models and treatment; **(B)** fecal quantities 10 min after intragastric administration; **(C)** time of first black feces after being intragastrically fed with ink on the day of sacrifice; **(D)** intestinal transport ratio (GI = ink movement length/total intestinal length*100%); **(E)** moving distances of each group of inks. NS; *p < 0.05; **p < 0.01; ***p < 0.001.

The overall experiment design was shown in Figure 1. After intervention, fecal quantities were measured 10 min after intragastric administration at a fixed time. Fecal quantities in the culture supernatant of *Lactobacillus paracasei* group (F) and hawthorn-probiotic group (FS) were significantly higher than those in the M group (Figure 1B). As demonstrated in Figure 1C, lactulose group (Y) presented the shortest time in term of the first black feces’ time after intragastrical administration with ink on the sacrifice day, followed by FS. Mice in F and hawthorn group (S) took longer time to defecate than mice in Y and FS group. The intestinal transport ratio in Y and FS group was significantly lower than that in the model group (M) (Figure 1D). The representative image of ink moving distance in each group was shown in Figure 1E.

### 2.2. Postbiotic of hawthorn-probiotic increased fecal water content by regulating water and sodium metabolism

**Figure 2:**
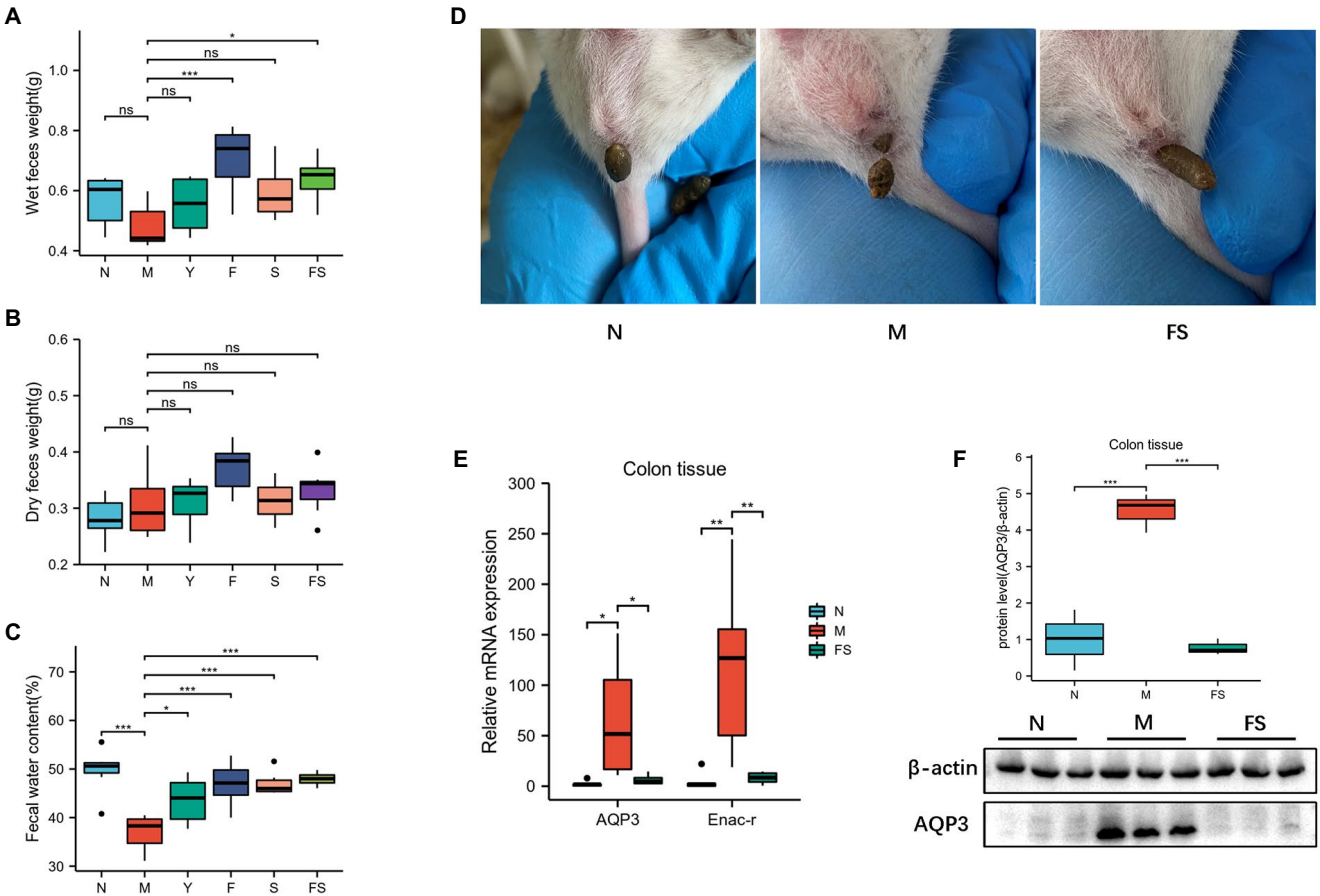
Postbiotic of hawthorn-probiotic maintain the stability of water and sodium metabolism and increase fecal water content. **(A)** Wet feces weight; **(B)** Dry feces weight; **(C)** Fecal water content (wet feces weight/dry feces weight); **(D)** Fresh feces in three groups of mice; **(E)** Relative mRNA expression of *AQP3* and *Enac-γ*; **(F)** AQP3 protein level. NS; *p < 0.05; **p < 0.01; ***p < 0.001.

The weight of fresh wet feces in F and FS group was heavier than that in M group (Figure 2A). But there were no differences in dry feces weight among these groups (Figure 2B). However, after calculating the fecal water content, we found that all the administration groups are better than the model group (Figure 2C). Combined with the above morphological changes, the effect of Postbiotic is the most obvious and stable. We used N, M, FS to explore the Postbiotic role. We can clearly see that the FS mice’s feces are more wet than those in M (Figure 2D).

We speculated that the above changes were induced by water and sodium metabolism changes. Then we tested the water and small-molecule channel aquaporin-3 (AQP3) and sodium channel epithelial 1 subunit gamma (Enac-γ) in different groups. It turned out that *AQP3* and *Enac-γ* in M were significantly increased than those in normal group (N). However, the expression of these two genes was decreased in FS (Figure 2E). Moreover, AQP3 protein had a similar trend to gene in the three groups (Figure 2F).

### 2.3. Postbiotic of hawthorn-probiotic preserved the intestinal barrier by promoting proliferation and reducing apoptosis

**Figure 3:**
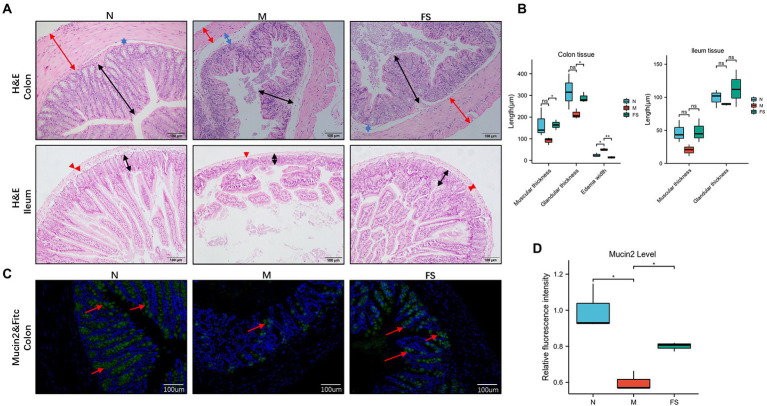
Postbiotic of Hawthorn-Probiotic help preserve the intestinal barrier. **(A)** Hematoxylin and eosin (H&E) staining in mice’s colon and ileum tissue; **(B)** muscle thickness, glandular thickness, edema width in colon; Muscle thickness, glandular thickness in ileum; **(C,D)** Mucin2 and FITC staining in colon tissue, Mucin2 is green, FITC is blue. NS; *p < 0.05; **p < 0.01; ***p < 0.001.

As shown in Figure 3A, the colonic and ileum’s muscular layer in M was thinner (red arrows), and the glands were fewer and atrophic (black arrows). Colonic hyperemia and edema in M were obvious, and the muscular layer was separated from the submucosa (blue arrows), tipping inflammation. Moreover, ileum villi in M showed obvious necrosis and exfoliation. The FS group represented remission on the above pathological changes. In Figure 3B, Postbiotic of Hawthorn-Probiotic (FS) is statistically significant on the increase in colonic muscle thickness and glandular thickness. M edema is more serious than N, and FS can effectively reduce edema.

**Figure 4:**
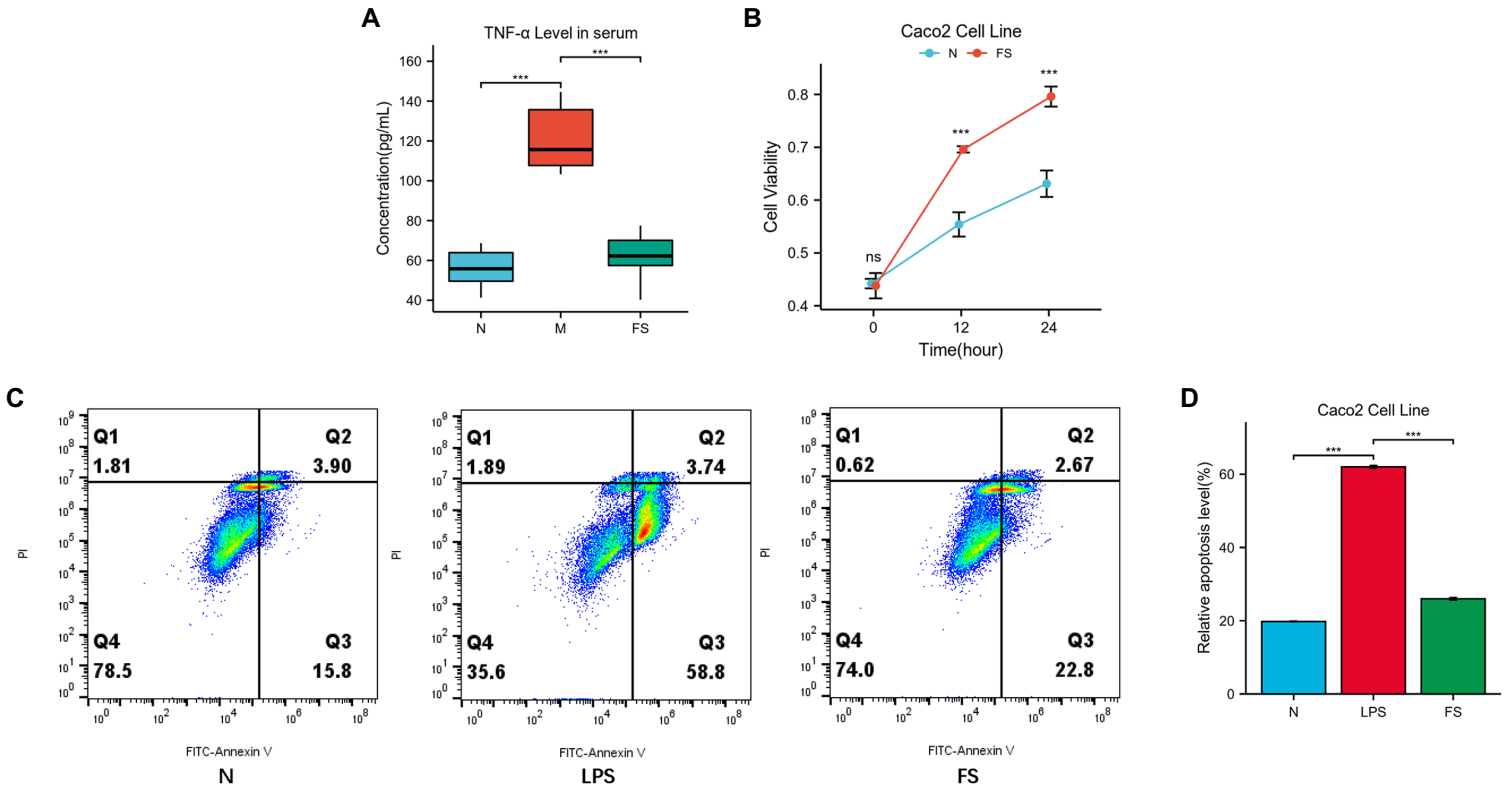
Postbiotic of hawthorn-probiotic help preserve the intestinal barrier. **(A)** The concentration of TNF-α in mice’s serum (pg/mL) by using Elisa; **(B)** Caco2 cells viability in normal and 5% postbiotic treated (FS) groups by using CCK8; **(C,D)** Caco2 cells relative apoptosis level (Q2 + Q3). NS; *p < 0.05; **p < 0.01; ***p < 0.001.

Mucin2 is a mucin mainly distributed in the digestive tract, secreted by goblet cells and glands. Its level is an important indicator of intestinal barrier function. As shown in Figure 3C, the level of mucin2 with green fluorescence in M was significantly decreased, tipping damage to the intestinal barrier. Then the mucin2 in FS was elevated than that in M (Figure 3D). We noticed that the intestinal cells in the FS group were fuller than those in the M group, with less necrosis and shedding. We speculated that these changes might be related to intestines inflammation since inflammation plays an important role in the pathogenesis of constipation, as demonstrated in Figure 3A. Therefore, we further tested serum TNFα of the constipated mice. It turned out that serum TNF-α was significantly elevated in M group and reversed in FS group (Figure 4A).

Since TNF-α is a key regulator and inducer of cell apoptosis, we speculated that apoptosis might be involved in intestinal barrier disruption. Then, Caco2 cell line was further applied to explore the mechanism by which postbiotic maintained the intestinal barrier. CCK-8 results showed that Caco2’s cell proliferation rates were significantly higher in 5% postbiotic than in 5% PBS at 12 and 24 h (Figure 4B). After 1 h of LPS stimulation, the 5% postbiotic culture for 24 h reduced the rate of early and late apoptosis of Caco2 cells (Figure 4C). Figure 4D showed that the apoptosis rate of FS was 3% (Q2 + Q3), while that of the LPS group was 7.5%.

### 2.4. Postbiotic of hawthorn-probiotic was safe and promoted blood circulation and spleen immune cell growth

**Figure 5:**
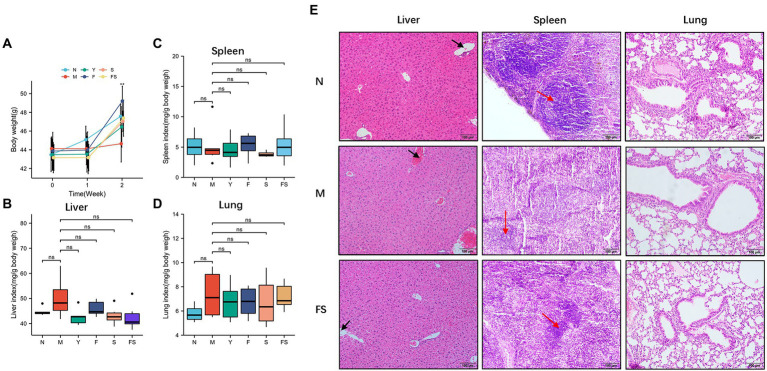
Postbiotic of hawthorn-Probiotic are safe and promote blood circulation and spleen immune cell growth. **(A)** Mice’s body weight after modeling and administration; **(B)** liver organ index of mice after administration; **(C)** spleen organ index of mice after administration; **(D)** lung organ index of mice after administration; **(E)** hematoxylin and eosin (H&E) staining in mice’s livers, spleens and lungs tissue. NS; *p < 0.05; **p < 0.01; ***p < 0.001.

Before and after modeling and administration, the mice in each group were weighed (Figure 5A). We found that the body weight of the mice was basically stagnated by loperamide modeling and recovered after administration.

We then used organ index, a relatively sensitive index of drug toxicity, to explore the safety performance of postbiotic of hawthorn-probiotic. We weighed the lungs, livers and spleens of mice and found there was no significant changes in organ index (Figures 5B–D). H&E staining was performed on the tissue sections. And we found that the liver of mice in M presented generally blood stasis, with the boundaries between the red pulp and the white pulp of the spleen blurred, and the distribution of immune cells were reduced compared with that of mice in N group. The above pathological changes were improved in FS group. No abnormalities were observed in the lungs.

### 2.5. Postbiotic of hawthorn-probiotic improved intestinal flora

**Figure 6:**
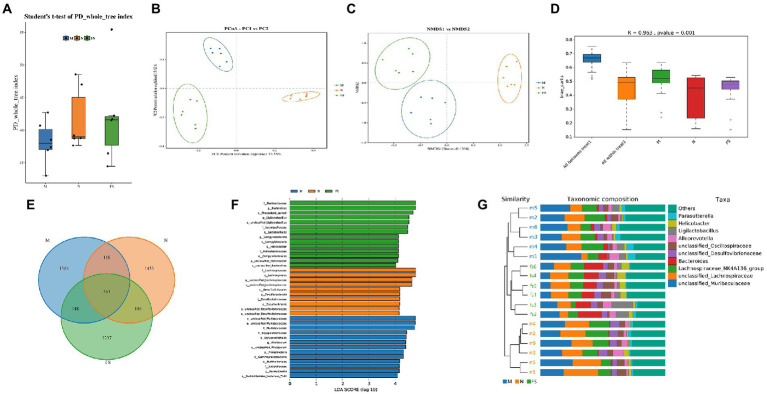
The structure of the three groups of bacteria is different. **(A)** PD whole tree in α-diversity; **(B)** principal coordinate analysis (pCoA) in *β*-diversity; **(C)** non-metric multiscale analysis (NMDS) in *β*-diversity; **(D)** anosim analysis; **(E)** Venn diagrams to compare the sequences of the three groups of bacteria; **(F)** histogram of LDA value distribution. The ordinate represents the taxonomic units with significant differences between groups, and the abscess represents the logarithmic score values of LDA analysis of each taxonomic unit in a bar chart. The taxon is sorted according to the size of the score value. The longer the length, the more significant the difference between the taxon is. The color of the bar graph indicates the sample group corresponding to the taxon with higher abundance; **(G)** phylogenetic tree and bar diagram to distinguish the three groups.

We conducted 16S rRNA to determine the changes of fecal microflora composition in the N, M and FS groups of mice. *α*- and *β*-diversity were used to explore the richness and evenness of gut microbiota. The results showed there was no statistical significance in terms of *α*-diversity among the three groups (Figure 6A). Principal coordinate analysis (pCoA) and non-metric multiscale analysis (NMDS) showed that the three groups were significantly clustered, with Stress = 0.1304 (less than 0.2 indicates the effectiveness of NMDS) (Figures 6B,C). Meanwhile, Anosim analysis showed that *R* = 0.963 (the closer it is to 1, the more effective the grouping is) (Figure 6D). Taken together, *β*-diversity analysis demonstrated that the gut flora patterns significantly differed among the three groups. By comparing the sequences of bacteria, we found that the microflora composition of the three groups was different (Figures 6E–G), but the structural composition within the groups was very similar. The intersection of common bacteria in the three groups was shown in Figure 6E. Three groups of bacteria with LDA > 4 are shown in Figure 6F. The result is similar to Figure 6G. The FS group was *Bacteroidaceae–Bacteroides-phocaeicola sartorii, Lactobacillales–Lactobacillaceae–Ligilactobacillus, Campylobacterales–Campylobacterota–Helicobacter* were mainly increased. According to the weighted phylogenetic tree, the bar diagram was formed by combining the bacteria groups. There was little difference within the three groups of bacteria, and they could be clearly distinguished from each other.

**Figure 7:**
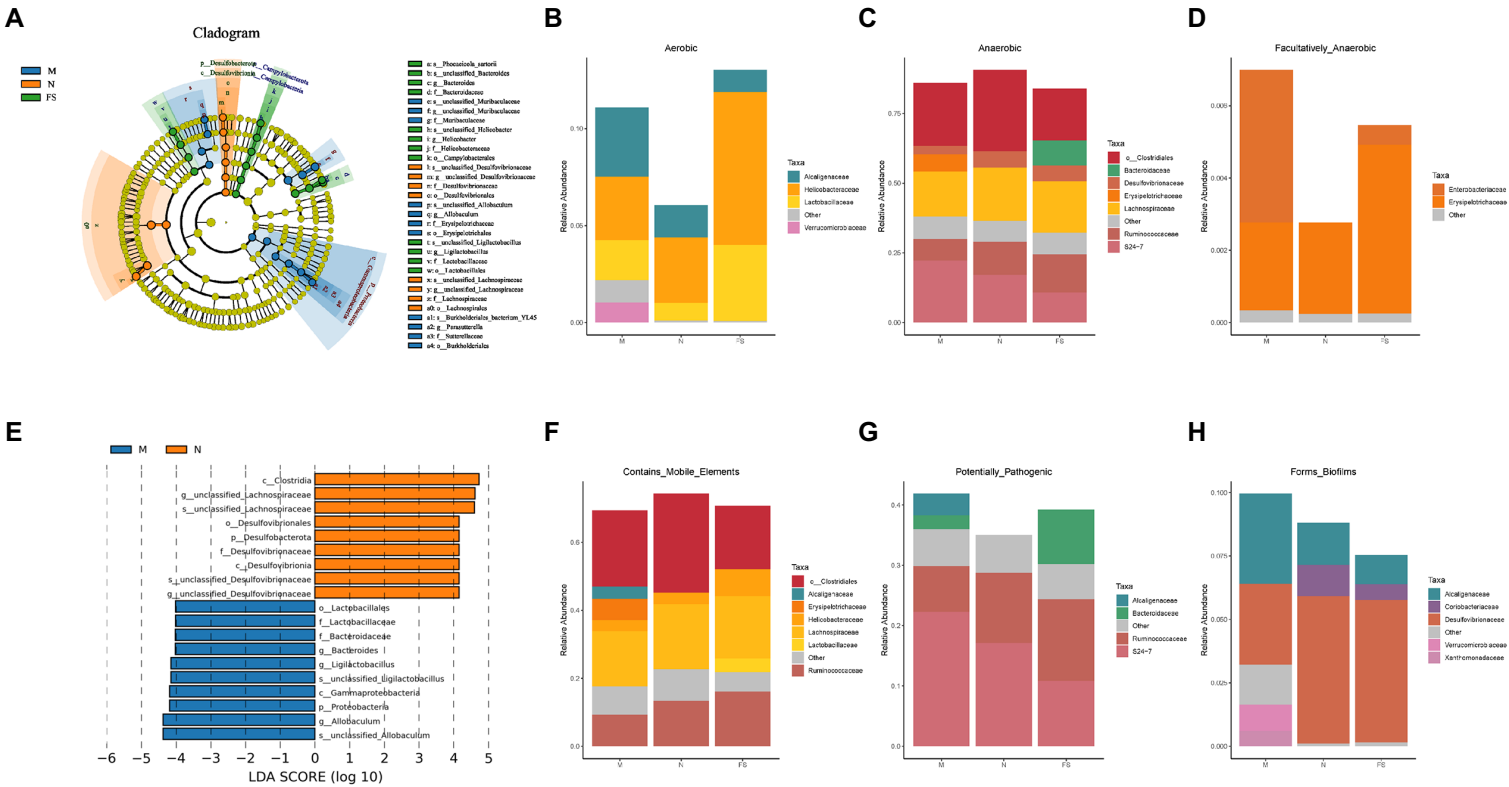
Postbiotic of hawthorn-probiotic decreases the abundance of intestinal or potential pathogens and increased the abundance of probiotics. **(A)** LEfSe analysis, the circles radiating from the inside out represent the classification levels from phylum to species; each small circle at a different classification level represents a classification at that level, and the diameter of the small circle is proportional to the relative abundance. The coloring principle is to color the species with no significant difference uniformly yellow, and color the other different species according to the group with the highest abundance. Different colors represent different groups, and different colored nodes represent the microbiome that plays an important role in the group represented by the color; **(B)** bar diagram of aerobic flora abundance at Family level in 3 groups; **(C)** bar diagram of the abundance of anaerobes at the Family level in 3 groups; **(D)** bar diagram of the abundance of facultative anaerobes at the Family level in 3 groups; **(E)** LDA analysis, the ordinate represents the classification units with significant differences between groups, and the abscess represents the logarithm score of LDA analysis of each classification unit in a bar graph. The classification unit is sorted according to the score value. The longer the length, the more significant the difference of the classification unit. The color of the bar graph indicates the sample group corresponding to the higher abundance of the classification unit; **(F)** bar diagram of the abundance of facultative contains mobile elements at the Family level in 3 groups; **(G)** bar diagram of the abundance of facultative potential pathogenic bacteria at the Family level in 3 groups; **(H)** bar diagram of the abundance of facultative bacteria who form biofilm at the Family level in 3 groups.

We identified the marker flora of the three groups from phylum, class, order, family, genus and species using LEfSe analysis (Figure 7A). Namely, *Desulfovibroides-Desulfovibrionaceae* and *Lachnospirales-Lachnospiraceae* were marker flora for N group, *Erysipelotrichales-Erysipelotrichaceae-Allobaculum*, *Burkholderiales-sutterellaceae-Parastutterella-Burkholderiales bacterium YL45*, and *Muribaculaceae* for M group, *Bacteroidaceae-Bacteroides-Phocaeicola sartorii*, *Campylobacterales-Helicobacteraceae-Helicobacter*, and *Lactobacillales-Lactobacillaceae-Ligilactobaillus* for FS group (the same to Figure 6E). *Proteobacteria* and *Firmicutes* were abundant in N group as in physical conditions and the most of flora in M group was pathogenic or potentially pathogenic. The decrease of the *Lactobacillales-Lactobacillaceae-Ligilactobaillus* could be used as the biomarker of M (LDA>4) (Figure 7E).

At the Family level, bacteria in the three groups were analyzed by abundance according to oxygen availability and three Features (Figures 7B–D,F). We found that microflora structure of FS and N was similar, and *Verrucomicrobiaceae* in M significantly increased, and postbiotic reduced the overall abundance of facultative anaerobes and reduced some bacteria who could form biofilms. It is well known that increased facultative anaerobic bacteria and biofilm formation of intestinal bacteria are associated with intestinal diseases.

We also analyzed data from three groups at the Genus level (Table 1). *Acetatifactor* significantly increased in M, but postbiotic did not increase it. Encouragingly, postbiotic sharply reduced the abundance of *Acinetobacter*, a kind of recognized pathogen. Puzzlingly, the abundance of *Akkermansia*, a beneficial bacterium in the gut, increased in M, which accounted for a small proportion in FS, and almost none in N. This may be related to the age and individual differences of the mice. *Alistipes*, producing short-chain fatty acids and reducing intestinal inflammation, increased in M, but did not change in FS. It may be a compensatory response to changes in the microbiome.

**Table 1:** ANOVA (analysis of variance) at genus level of bacterial microbiota.

Genus	*M* (mean)	*M* (se)	*N* (mean)	*N* (se)	*T* (mean)	*T* (se)	Multigroup (*p*)
A2	0.000264	0.000159	0.000859	0.000301	0.000447	0.000225	0.221101
ASF356	0.001374	0.000253	0.001325	0.000227	0.002247	0.000722	0.309511
**Acetatifactor**	0.000099	0.000099	0.001188	0.000436	0.000026	0.000026	**0.010101**
**Acinetobacter**	0.004912	0.001975	0.000017	0.000008	0.000006	0.000006	**0.011175**
Actinomadura	0.000000	0.000000	0.000000	0.000000	0.000006	0.000006	0.391127
Aerococcus	0.000009	0.000009	0.000000	0.000000	0.000000	0.000000	0.391127
Aeromonas	0.000000	0.000000	0.000000	0.000000	0.000016	0.000016	0.391127
Agathobacter	0.000000	0.000000	0.000011	0.000011	0.000000	0.000000	0.391127
**Akkermansia**	0.005916	0.001222	0.000000	0.000000	0.000121	0.000111	**0.000029**
**Alistipes**	0.012515	0.001609	0.006836	0.001133	0.011167	0.001220	**0.022686**

### 2.6. Postbiotic of hawthorn-probiotic may promote intestinal microbiotic restoration and relieve constipation by reducing pathogenic bacteria and increasing cell activity

**Figure 8:**
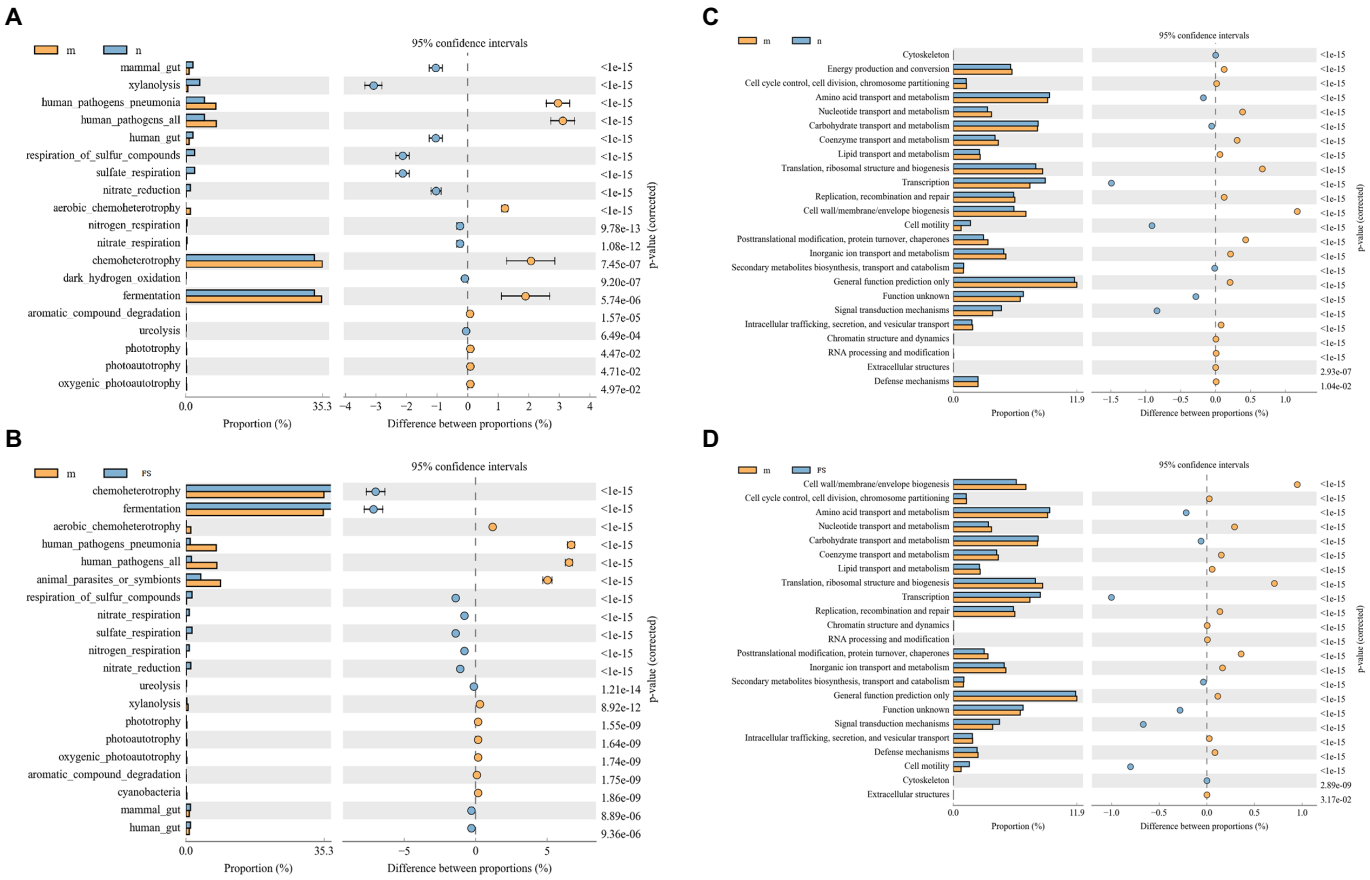
Postbiotic of hawthorn-probiotic reduces the pathogenic microbial pathway and increases the cell activity pathway by enrichment analysis. **(A)** The FAPROTAX Ecological function forecast between M and N; **(B)** the FAPROTAX Ecological function forecast between M and FS; **(C)** COG functional classification statistical chart between M and N; **(D)** COG functional classification statistical chart between M and FS. The figure on the left shows the abundance ratio of different functions in two samples or groups of samples. In the middle is the proportion of differences in functional abundance within the 95% confidence interval. The rightmost value is a value of *p*.

According to the predicted enrichment results, we find that pathogenic bacteria and parasite-related pathways were elevated in M (Figure 8A), while improvements were observed in FS (Figure 8B). Moreover, the cell activity of M was decreased (Figure 8C), and the cell activity was increased after administration (Figure 8D).

## 3. Discussion

Constipation, as a common gastrointestinal disease, troubles people, especially the elderly. Dietary modification instead of medication is reckoned as optimal therapy for constipated patients. In our study, with loperamide modeled elderly KM mice, we found that postbiotic of hawthorn-probiotic exerted remarkable curative effects, without toxicity and side effects. It might attenuate the water and sodium metabolism, chronic inflammation of the intestine, accompanied by promoted cell proliferation and reduced cell apoptosis. Besides, it could maintain the intestinal microecology by repressing the harmful bacteria colonization and improving the abundance of probiotics.

Water and sodium metabolism, intestinal inflammation are interacting factors during the disease course of constipation, which participate in intestinal motility and the balance of intestinal barrier (27–29). AQP3 is a water channel protein required to promote glycerol permeability and water transport across cell membranes (30). There is increasing evidence demonstrating that AQP3 in involved in inflammatory diseases including atopic dermatitis, psoriasis, allergy, and cancer progression, using AQP3^−/−^ mice and AQP3-knockdown cells (31–33). Enac-γ is a sodium permeable non-voltage-sensitive ion channel inhibited by the diuretic amiloride, mediating the electro-diffusion of the luminal sodium (and water, which follows osmotically) through the apical membrane of epithelial cells (34). It plays an essential role in electrolyte and blood pressure homeostasis, but also in airway surface liquid homeostasis, which is important for proper clearance of mucus (35). And it also controls the reabsorption of sodium in kidney, colon, lung and sweat glands, also plays a role in taste perception (34, 35). These findings suggest that when Enac-γ is out of balance, it can cause electrolyte disturbances, blood pressure fluctuations, and changes in colon mucus. Mucin2 (MUC2) coats the epithelia of the intestines, airways, and other mucus membrane-containing organs. It is thought to be protective, lubricating barrier against particles and infectious agents at mucosal surfaces (36). Major constituent of both the inner and outer mucus layers of the colon may play a role in excluding bacteria from the inner mucus layer (36).

Combined with various indexes of elderly KM mice in group M, we analyzed that intestinal motility decreased and fecal retention occurred in the colon during constipation. The expression of *AQP3* and *Enac-γ* increased, and the metabolism of water and sodium increased. With increased intestinal permeability, water, mucus and sodium were absorbed in large quantities, and fecal water content decreased. The whole-body may have electrolyte disorders, blood pressure abnormalities, so the liver blood flow was not smooth. Intestinal pathogenic bacteria stimulate intestinal epithelial cells and destroy intestinal epithelial structure. Mucin2 is decreased in goblet cells and glands of the large intestine. Glandular atrophy, mucosal layer and epithelial cell shedding and necrosis reflect decreased intestinal barrier function. Pathogenic bacteria increase, immune cell infiltration, secretion of TNF-α, intestinal mucosal epithelium accelerated necrosis and shedding, also promote intestinal congestion and edema. The gut is in a state of inflammation. We found postbiotic of Hawthorn-Probiotic can effectively reduce water and sodium metabolism, maintain intestinal tissue integrity, restore intestinal barrier, and reduce inflammatory factors.

The gut is home to trillions of microbes, and their changes indicate an imbalance of homeostasis of the body. The most immediate changes are local functional and organizational changes in the gut, and this is also an important factor in the occurrence of constipation (37). There are many reports about the changes of intestinal microflora in constipation and the recovery of microflora after treatment (8, 38–40). The abundance of pathogenic bacteria and potential pathogenic bacteria increased in the disease state, the number of probiotics decreased, and improved after treatment. Our results not only showed the increase of pathogenic bacteria (*Acinetobacter*, *Erusipelotrichaceae*, etc.) in the model group, but also the increase of pathogenic bacteria forming biofilm. Biofilms have been found in a variety of intestinal diseases (tumor, inflammatory bowel disease, colitis, etc.) and are important factors in the development of drug resistance (41–45). When bacteria form biofilms, resistance can increase by up to 1,000 times (43, 46). The increased biofilm of intestinal bacteria in constipated mice is a bad phenomenon. Among these bacteria increasing biofilm, *Xanomonadaceae* is a kind of pathogenic bacteria that secretes effector proteins to enhance the adaptability of bacteria to the external environment and mediate the virulence of bacteria to the host (47). *Verrucomicrobiaceae*, a newly delineated group of bacteria that includes a handful of recognized species, has been found mostly in aquatic and soil environments, or in human feces (48). It was discovered relatively recently. At present, the research on it mainly focuses on the changes of the flora of various diseases. And the change is irregular. So it’s impossible to infer its exact function, good or evil. It is certain that the formation of biofilms by these bacteria increases, boding ill for the future. Excitingly, this phenomenon in constipation is first reported by us. It also indicates that deserves more attention. What is more shocking to us is that postbiotic of hawthorn-probiotic can treat the biofilm and adjust the structure of the flora to a normal state. And then we are going to do more *in vitro* studies to investigate this phenomenon.

*Bacteroides* is the main bacteria that can decompose polysaccharides in human body (49–51). It produces short chains and organic acids that can be absorbed by the host (52). The increase of Bacteroides in FS may be caused by polysaccharides in hawthorn. *Lactobacillus* is a well-known probiotic, and it has a wide variety and is widely used. The increase of *Lactobacillus* should be a direct effect of *Postbiotic*. *Akkermansiamuciniphila* (*Akk*) is a kind of normal bacteria in human intestinal tract (53), which is a mucin-degrading bacteria (54, 55). The strain MucT was isolated by Derrien in 2004 (56). It is an ovoid, gram-negative anaerobe and a representative of the phylum vermicella. However, there was no *Akk* in normal old KM mice, which we thought might be related to the differences of age and individual mice. As for why there is *Akk* in M and FS, there are more M, which we analyze that reduction of intestinal MUC2 was associated. Similar to the increase of AQP3, *Akk* may increase mucin absorption and decomposition, reduce MUC2 content, and reduce intestinal barrier function. After *Postbiotic* treatment, *Akk* showed a downward trend.

## 4. Study limitations

Due to limited conditions, we did not perform metabonomics analysis.

Caco2 cell line is commonly used to study the potential of drug absorption, the mechanism of drug transport (absorption and elimination mechanism), and the intestinal metabolism of drugs, nutrients and plant components. It’s okay for us to experiment with it. But it’s also a cancer cell, and we are missing that. Due to limited condition, we did not carry out the extraction and experiment of primary cells.

We found an increase in TNF-α in the serum of the constipated mice, but local inflammation of the colon was not validated due to the absence of intestinal tissue.

## 5. Materials and methods

### 5.1. Key resources table

**Table:** 

Reagent or resource	Source	Identifier
**Antibodies**		
AQP3	Affinity	DF6127
β-actin (13E5)	CST	4970S
Mucin2	Servicebio	AF5222
goat anti-rabbit IgG secondary antibody (1:2000)	Servicebio	G2210-2-A
Alexa Fluor 488-conjugated Goat Anti-Rabbit IgG	Servicebio	GB25303
**Chemicals**		
Blood agar plates (TSA with 5% sheep blood)	Thermo Fisher	R02050
Radio immunoprecipitation assay (RIPA) lysis buffer	Thermo Scientific	78,510
horseradish peroxidase- conjugated goat anti-mouse	Servicebio	G2211-1-A
EDTA antigen retrieval solution (pH 8.0)	Servicebio	G1206
anti-fade mounting medium	Servicebio	G140
Liquid blocker pen	Servicebio	G6100
Spontaneouss fluorescence quenching reagent	Servicebio	G1221
Penicillin–streptomycin solution	CORNING	30,002,304
Phosphate-buffered saline (PBS)	CORNING	19,117,004
Fetal bovine serum	Gibco	1,932,595
0.25% Trypsin–EDTA (1×)	Gibco	2,042,337
DMEM basic (1×)	Gibco	8,119,090
Loperamide (imodium)	Xian Janssen Pharmaceutical Ltd.	LGJ0549
Lactulose (Duphalac)	Abbott	H20171057
Lipopolysaccharide (LPS)	Sigma-Aldrich	L2630
Critical commercial assays		
Annexin V-FITC/PI	Melunbio	MA0229
Mouse TNF-alpha ELISA Kit	Proteintech	KE10002
CCK-8	Solarbio	CA1210
RNAiso Plus	Vazyme	R401
HiScriptII Q RT SuperMix for qPCR (+gDNA wiper)	Vazyme	R223
ChamQ SYBR qPCR Master Mix	Vazyme	Q311
Software and algorithms		
R version3.6.3	The R Foundation	N/A
Image J	NIH	N/A
FlowJo V10	BD	N/A
SIMCA	Umetrics AB	N/A
Sequence of primers		
Genes	Sequence of primers	
*AQP3*	5′-CGCTGGTGTCTTCGTGTACC-3’	
	5′-TGTGGGCCAGCTTCACATTC-3’	
*Enac-γ*	5’-TGAGTGACCTCCTGACTGACTTGG-3’	
	5’-GAAATCTGGGTGGTGTGCCTTCC-3’	
Universal bacterial primers	5’-AGAGTTTGATCCTGGCTCAG-3’	
	5’-GGTTACCTTGTTACGACTT-3’	

### 5.2. Preparation of hawthorn aqueous extract and postbiotic

Ten gram caseinase digestion, 10 g beef paste powder, 4 g yeast paste powder, 2 g triammonium citrate, 5 g sodium acetate, 0.2 g magnesium sulfate, 0.05 g manganese sulfate, 2 g dimethyl hydrogen phosphate, 20 g glucose and 1.08 g Tween 80 were added into each liter of distilled water. Adjust the final pH value of this liquid to 5.7 ± 0.2. Then autoclave it, 121°C for 20 min. The monoclonal *Lactobacillus paracasei* (isolated from baby feces, identified again by colony PCR, the sequence was shown in Supplementary Table S1) cultured on the blood agar plates was added into it. Then place it in a shaker at 35°C and 220 rpm for 8 h. The OD600 value measured was 0.8–1.2. Some of the liquids was retained, and the remaining was continued in a shaker at 35°C and 220 rpm for 72 h. The supernatants were centrifuged at 12,000 *g* for 10 min. Filter it with a 0.22-μm filter. The hawthorns were soaked in distilled water for 0.5 h, and then boiled at 100°C for 30 min. The liquids were concentrated to 1 g/ml and then filtered with a 0.22-μm filter. The retained liquids were centrifuged with 8,000 g for 10 min to obtain the bacteria. Then the sterile hawthorn liquids were poured into the bacteria container. After fermentation for 72 h in a shaker at 35°C and 220 rpm, the supernatants were centrifuged at 12,000 *g* for 10 min and filtered with a 0.22-μm filter to obtain the postbiotic of hawthorn-probiotic.

### 5.3. Animals and groups

Two hundred and forty days old, male KM mice obtained from the Guangzhou Regal Biotechnology Co., Ltd., (SCXK [Yue] 2018–0182, SYXK [Yue] 2021–0059) were pair-housed in plastic cages in a temperature-controlled (25 ± 2°C) colony room under a 12/12-h light/dark cycle, with free access to food and water. All experimental protocols were approved by the Animal Center, Guangzhou University of Chinese Medicine.

The aged KM male mice were administrated with distilled water throughout the whole course and 6 mice were grouped as the normal controls without any other intervention (N). The rest mice were treated with 5 mg/kg loperamide for 1 week and randomly divided into model group (M), positive drug group (Y), hawthorn group (S), probiotic group (F) and postbiotic of hawthorn-probiotic group (FS). Mice in Y group were intragastrically treated with 10% lactulose (0.2 ml/day/per mouse), S group with 1 g/ml pure hawthorn solution (0.2 ml/day/per mouse), F group with *Lactobacillus paracasei* supernatant (0.2 ml/day/per mouse) and FS group with postbiotic of hawthorn-probiotic (0.2 ml/day/per mouse) for another week. The body weight and other health indexes, fecal water content, intestinal propulsion rate, the levels of inflammatory factors *in vivo*, the structure of ileum and colon, and fecal flora of mice were determined.

### 5.4. Histopathology and immunofluorescence

The lung, spleen, liver, ileum, and colon tissues were removed and fixed in 4% paraformaldehyde at pH 7.4 for further pathological experiments. These tissue samples were then embedded in paraffin and cut into 4 μm sections. Sections were stained with hematoxylin–eosin (H&E). Slides were observed under a light microscope.

Deparaffinize and rehydrate: incubate sections in 3 changes of Biodewax and Clear Solution, 10 min each. Dehydrate in 3 changes of pure ethanol for 5 min. Wash in distilled water. Antigen retrieval: immerse the slides in EDTA antigen retrieval buffer (pH 8.0) and maintain at a sub-boiling temperature for 8 min, standing for 8 min and then followed by another sub-boiling temperature for 7 min. Be sure to prevent buffer solution evaporate. Let air cooling. Wash three times with PBS (pH 7.4) in a Rocker device, 5 min each. Use the right antigen retrieval buffer and heat extent according to tissue characteristics. Circle and Serum blocking: eliminate obvious liquid, mark the objective tissue with liquid blocker pen. Add 3% BSA to cover the marked tissue to block non-specific binding for 30 min. Cover objective area with 10% donkey serum (for the case of primary antibody originated from goat) or 3% BSA (for the case of primary antibody originated from others). Primary antibody: throw away the blocking solution slightly. Incubate slides with primary antibody (diluted with PBS appropriately) overnight at 4°C, placed in a wet box containing a little water. Secondary antibody: wash slides three times with PBS (pH 7.4) in a Rocker device, 5 min each. Then throw away liquid slightly. Cover objective tissue with secondary antibody (appropriately respond to primary antibody in species), incubate at room temperature for 50 min in dark condition. FITC counterstain in nucleus: wash three times with PBS (pH 7.4) in a Rocker device, 5 min each. Then incubate with FITC solution at room temperature for 10 min, kept in dark place. Spontaneous fluorescence quenching: wash three times with PBS (pH 7.4) in a Rocker device. 5 min each. Add spontaneous fluorescence quenching reagent to incubate for 5 min. Wash in running tap water for 10 min. Mount: Throw away liquid slightly, then cover slip with anti-fade mounting medium. Microscopy detection and collect images by Fluorescent Microscopy.

### 5.5. RNA isolation and quantitative analysis (RT-qPCR)

RNA was extracted from colon tissue using RNAiso Plus according to the instructions. Then, cDNA was obtained using the ImProm-II™ Reverse Transcription System (Promega) and RT-qPCR was carried out with custom designed oligonucleotides using the HiScriptII Q RT SuperMix for qPCR (+gDNA wiper) and the ChamQ SYBR qPCR Master Mix in a total volume of 20 μl:95°C for 1 min and 40 cycles of denaturation (95°C for 15 s) and extension (60°C for 1 min). Experiments were performed in triplicates. Following amplification, dissociation curve analyses were performed to confirm the amplicon specificity for each PCR run. The relative level of gene expression in mouse colon tissue was normalized against mouse *β*-actin, respectively. Analysis of relative expression was performed using the 2^(−ΔΔCT)^ method.

### 5.6. Western blotting

Briefly, global colon tissue was dissected from treated mice and proteins extracted with radioimmunoprecipitation assay (RIPA) lysis buffer. The proteins were separated by sodium dodecyl sulfate-polyacrylamide gel electrophoresis and transferred onto polyvinylidene fluoride membranes. After blocking with 5% nonfat dry milk in Tris-buffered saline (20 mM Tris–HCl, 500 mM NaCl, pH 7.4) with 0.2% Tween-20, the membranes were probed with antibodies overnight at 4°C, followed by incubation with a horseradish peroxidase- conjugated goat anti-mouse or goat anti-rabbit IgG secondary antibody (1: 2000). Band intensity was quantified using ImageJ software (NIH).

### 5.7. Cell culture

Caco2 cells were obtained from the National Collection of Authenticated Cell Cultures. Cells were cultured in DMEM Medium with 10% fetal bovine serum and 1% Penicillin–streptomycin solution as routine. All cells were grown in a humidified incubator at 37°C with 5% CO2.

### 5.8. CCK-8 detection

Eight thousand cells per well of Caco2 cells suspension was prepared in a 96-well plate. The control group’s (N) wells were added with ordinary cell medium containing 5% PBS to 100 μl per well. In the experimental group (FS), cell mediums containing 5% postbiotic were added to 100 μl per well. Replace the fresh cell culture medium and add 10 μl of CCK-8 solution to each well in 12 h and 24 h.

### 5.9. Flow cytometry with the Tdt-mediated UTP nick-end labeling

1,000,000 cells per well of Caco2 cells suspension was prepared in a 6-well plate. After 24 h, the cells adhere. Replace the fresh cell culture medium in control group’s (N) wells. And replace cell medium containing 10 ng/ml LPS in Model (LPS) and experimental group’s (FS) wells. After 1 h, discard the two group’s medium and use PBS washing wells for three times. Replace the fresh cell culture medium in LPS’s wells. Replace the cell medium containing 5% postbiotic in FS’s wells. After 24 h, wash all wells with PBS. Add 500 μl trypsin to each well. After 2 min, add 1 ml medium to each well. Centrifuge at 2000 rpm for 5 min. Discard the medium. Add 100 μl binding buffer (1X), 5 μl FITC-Annexin V, 5 μl PI for each sample. After 15 min, cell apoptosis was detected by flow cytometry.

### 5.10. Bioinformatics analysis of 16 S rRNA from fecal bacteria

Sequences of the V3-V4 region of 16S rRNA genes were detected using an Illumina HiSeq 2,500 platform (Biomarker Technology Co. Ltd., Beijing, China). OTUs present in 50% or more of the fecal samples were identified as core OTUs. The observed species, Shannon, Simpson and PD whole tree were calculated with QIIME2 2020.6 (57, 58). The abundance and diversity of the OTUs (beta diversity) were examined using principal-coordinate analysis (PCoA) with weighted UniFrac analysis in R software. The linear discriminant analysis (LDA) effect size analysis (LEfSe) was used with the *Kruskal–Wallis* rank sum test to detect features with significantly different abundances between assigned taxa, and linear discriminant analysis was performed to estimate the effect size of each feature. The bacterial groups with LDA score of 4.00 were presented as the significantly abundant group in the indicated group. The phylogenetic tree was constructed between the feature sequences (16S rRNA) and the Integrated Microbial Genome Database (IMG) Reference sequence alignment (aliign) using PICRUSt2, and the “nearest species” of the feature sequences were found. The gene information of unknown species was predicted based on the information of gene type and abundance of known species, and the pathway of the whole community was predicted based on the KEGG pathway information of genes. COG (Clusters of Orthologous Groups of proteins) was a commonly used protein functional classification database of prokaryotes. COG function prediction analysis method was basically the same as KEGG. Faprotax (59) forecast analysis was conducted according to the literature.

## Data availability statement

The 16S sequencing datasets presented in this study can be found in NCBI. The accession number(s) is PRJNA939336. Other data has been aggregated into the Supplementary material.

## Ethics statement

The animal study was reviewed and approved by Experimental Animal Ethics Committee, Guangzhou University of Chinese Medicine.

## Author contributions

YH conceived and designed the study, coordinated the study, and obtained the fund. YW, NH, and ML conducted the experiments, performed the data collection and analysis. XY and MW were responsible for raising the mice. YW drafted the manuscript. YH critically revised the manuscript. All authors have read and approved the final manuscript.

## Funding

This research was funded by grants from Chinese National Natural Science Foundation (Grant No. 31700288).

## Conflict of interest

The authors declare that the research was conducted in the absence of any commercial or financial relationships that could be construed as a potential conflict of interest.

## Publisher’s note

All claims expressed in this article are solely those of the authors and do not necessarily represent those of their affiliated organizations, or those of the publisher, the editors and the reviewers. Any product that may be evaluated in this article, or claim that may be made by its manufacturer, is not guaranteed or endorsed by the publisher.
