# Supplementary material for: The postbiotic of hawthorn-probiotic ameliorating constipation caused by loperamide in elderly mice by regulating intestinal microecology
Source: Front Nutr. 2023 Mar 16;10:1103463. doi: 10.3389/fnut.2023.1103463 (PMC10061020; doi:10.3389/fnut.2023.1103463)
Supplement: Supplementary file 1 [file Data_Sheet_1.zip › supply materials/Animal Ethics Table.pdf]

申请单号：20221031002

广州中医药大学动物实验伦理审查结果

申请日期：2022年10月31日

课题名称	副干酪乳杆菌中药发酵上清治疗慢性便秘的作用及机制				
申请人	韦钰	职称/学位	硕士	邮箱	623006899@qq.com
课题负责人	黄雅丽	职称/学位	副教授	邮箱	avonlii@163.com
院系（部门）	基础医学院				
动物种系	小鼠，KM	数量	40		
审查依据	1、该项目是否必须用实验动物进行实验，即能否用计算机模拟、细胞培养等非生命方法替代动物或用低等动物替代高等动物进行实验。<br>2、表中所填实验相关人员资格和实验相关单位是否合适。<br>3、表中所填实验所用动物能否通过改良设计方案或用高质量的动物来减少所用动物的数量。<br>4、能否通过改进实验方法、调整实验观测指标、改良处死动物的方法，来优化实验方案、善待动物 。<br>5、实验设计、实验技术方法及用于本实验的动物数量是否合理可行。				
伦理委员审查意见	经审查，课题设计符合《广州中医药大学动物实验伦理审查条例》要求，同意开展动物实验				
伦理委员	骆欢欢	审查日期	2022年11月04日		
学校实验动物伦理委员会审查意见					
主任委员		审查日期			

说明：

1、 必须写明所有实验参与人员，参与人员应取得动物实验培训证书（如是学生，必须修读过医学实验动物学课程并考试合格）。

2、 课题负责人必须保证所填资料的真实性，如实验过程中违反动物福利、伦理要求，将被清退出实验室。
