# Supplementary material for: The postbiotic of hawthorn-probiotic ameliorating constipation caused by loperamide in elderly mice by regulating intestinal microecology
Source: Front Nutr. 2023 Mar 16;10:1103463. doi: 10.3389/fnut.2023.1103463 (PMC10061020; doi:10.3389/fnut.2023.1103463)
Supplement: Supplementary file 1 [file Data_Sheet_1.zip › supply materials/Animal qualification certificate.pdf]

# 广东省实验动物质量合格证明

No.44827200002530

购买单位：广州中医药大学（中西医结合基础研究中心）　　动物实验单位：广州中医药大学（中西医结合基础研究中心）

| 动物品种品系 | 等级 | 动物规格 | | 数量 |
|---|---|---|---|---|
| | | 体重/日龄 | 性别 | |
| 小鼠, KM | SPF级 | 240天 | 雄性 | 40 |
| 最近一次质量检测日期 | 2022年07月18日 | | 质量检测单位 | 广东省实验动物监测所 |
| 用途 | 科学研究 | | 实验单位使用许可证编号 | SYXK（粤）2018-0182 |
| 出售单位(盖章) | 广州锐格生物科技有限公司 | | 许可证号 | SCXK（粤）2021-0059 |

质量负责人：王全捷　　经手人：王艳菲　　日期：2022年10月18日


# 广东省实验动物质量合格证明

No.44827200002530

购买单位：广州中医药大学（中西医结合基础研究中心）　　动物实验单位：广州中医药大学（中西医结合基础研究中心）

| 动物品种品系 | 等级 | 动物规格 | | 数量 |
|---|---|---|---|---|
| | | 体重/日龄 | 性别 | |
| 小鼠, KM | SPF级 | 240天 | 雄性 | 40 |
| 最近一次质量检测日期 | 2022年07月18日 | | 质量检测单位 | 广东省实验动物监测所 |
| 用途 | 科学研究 | | 实验单位使用许可证编号 | SYXK（粤）2018-0182 |
| 出售单位(盖章) | 广州锐格生物科技有限公司 | | 许可证号 | SCXK（粤）2021-0059 |

质量负责人：王全捷　　经手人：王艳菲　　日期：2022年10月18日
