# Supplementary material for: The postbiotic of hawthorn-probiotic ameliorating constipation caused by loperamide in elderly mice by regulating intestinal microecology
Source: Front Nutr. 2023 Mar 16;10:1103463. doi: 10.3389/fnut.2023.1103463 (PMC10061020; doi:10.3389/fnut.2023.1103463)
Supplement: Supplementary file 1 [file Data_Sheet_1.zip › supply materials/Supplementary Material in manuscript.docx]

# Supplemental Materials

## 1.Supplemental tables

| Results of strain identification | Sequence |
| --- | --- |
| *Lactobacillus paracasei subsp. tolerans* | GGGGGCATGGCGGCGTGCTATACATGCAAGTCGAACGAGTTCTCGTTGATGATCGGTGCTTGCACCGAGATTCAACATGGAACGAGTGGCGGACGGGTGAGTAACACGTGGGTAACCTGCCCTTAAGTGGGGGATAACATTTGGAAACAGATGCTAATACCGCATAGATCCAAGAACCGCATGGTTCTTGGCTGAAAGATGGCGTAAGCTATCGCTTTTGGATGGACCCGCGGCGTATTAGCTAGTTGGTGAGGTAATGGCTCACCAAGGCGATGATACGTAGCCGAACTGAGAGGTTGATCGGCCACATTGGGACTGAGACACGGCCCAAACTCCTACGGGAGGCAGCAGTAGGGAATCTTCCACAATGGACGCAAGTCTGATGGAGCAACGCCGCGTGAGTGAAGAAGGCTTTCGGGTCGTAAAACTCTGTTGTTGGAGAAGAATGGTCGGCAGAGTAACTGTTGTCGGCGTGACGGTATCCAACCAGAAAGCCACGGCTAACTACGTGCCAGCAGCCGCGGTAATACGTAGGTGGCAAGCGTTATCCGGATTTATTGGGCGTAAAGCGAGCGCAGGCGGTTTTTTAAGTCTGATGTGAAAGCCCTCGGCTTAACCGAGGAAGCGCATCGGAAACTGGGAAACTTGAGTGCAGAAGAGGACAGTGGAACTCCATGTGTAGCGGTGAAATGCGTAGATATATGGAAGAACACCAGTGGCGAAGGCGGCTGTCTGGTCTGTAACTGACGCTGAGGCTCGAAAGCATGGGTAGCGAACAGGATTAGATACCCTGGTAGTCCATGCCGTAAACGATGAATGCTAGGTGTTGGAGGGTTTCCGCCCTTCAGTGCCGCAGCTAACGCATTAAGCATTCCGCCTGGGGAGTACGACCGCAAGGTTGAACTCAAAGGAATTGACGGGGGCCCGCACAAGCGGTGGAGCATGTGGTTTAATTCGAGCACCGCGAGACCCTACCAGTCTTGACATCTTTTGATCACCTTGAGAGATCAGGTTTCCCCTTCCGGGGCAAATGACAGGTGGTGCATGTTGTTCGTCAGCCTCGTGTCGTGAGATGTTGGGTAGTTCCCGCAACGAAGCGCAACCTCATATAG |

Table S1: 16s rRNA sequence of *Lactobacillus paracasei*.

## 2.Supplemental figures


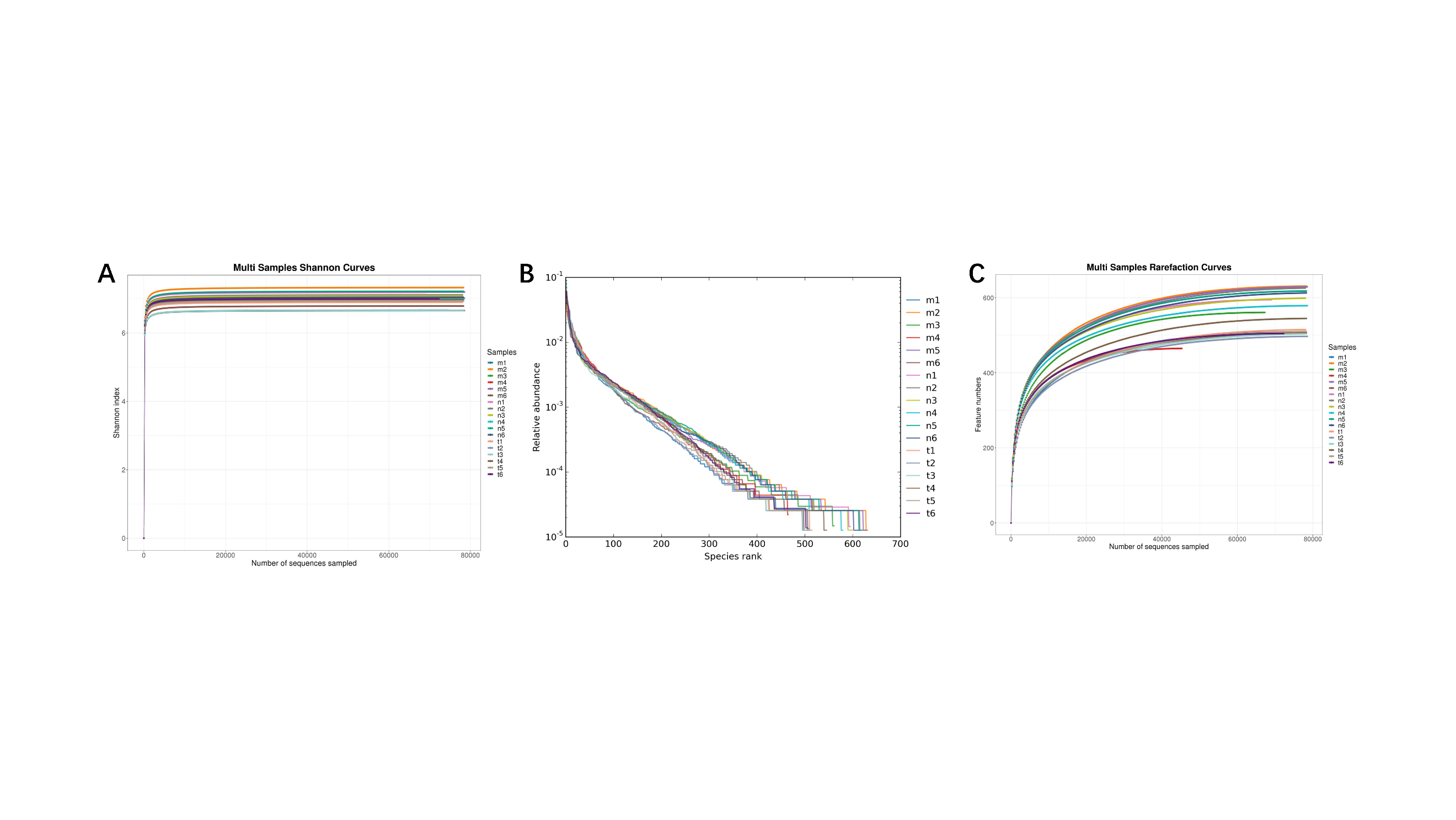


Figure S1: Depth of 16s rRNA sequencing. (A)Shannon Curves; (B) Sparse distribution line graph; (C) Abundance grade curve.
