# Supplementary figures and images for: The postbiotic of hawthorn-probiotic ameliorating constipation caused by loperamide in elderly mice by regulating intestinal microecology
Source: Front Nutr. 2023 Mar 16;10:1103463. doi: 10.3389/fnut.2023.1103463 (PMC10061020; doi:10.3389/fnut.2023.1103463)

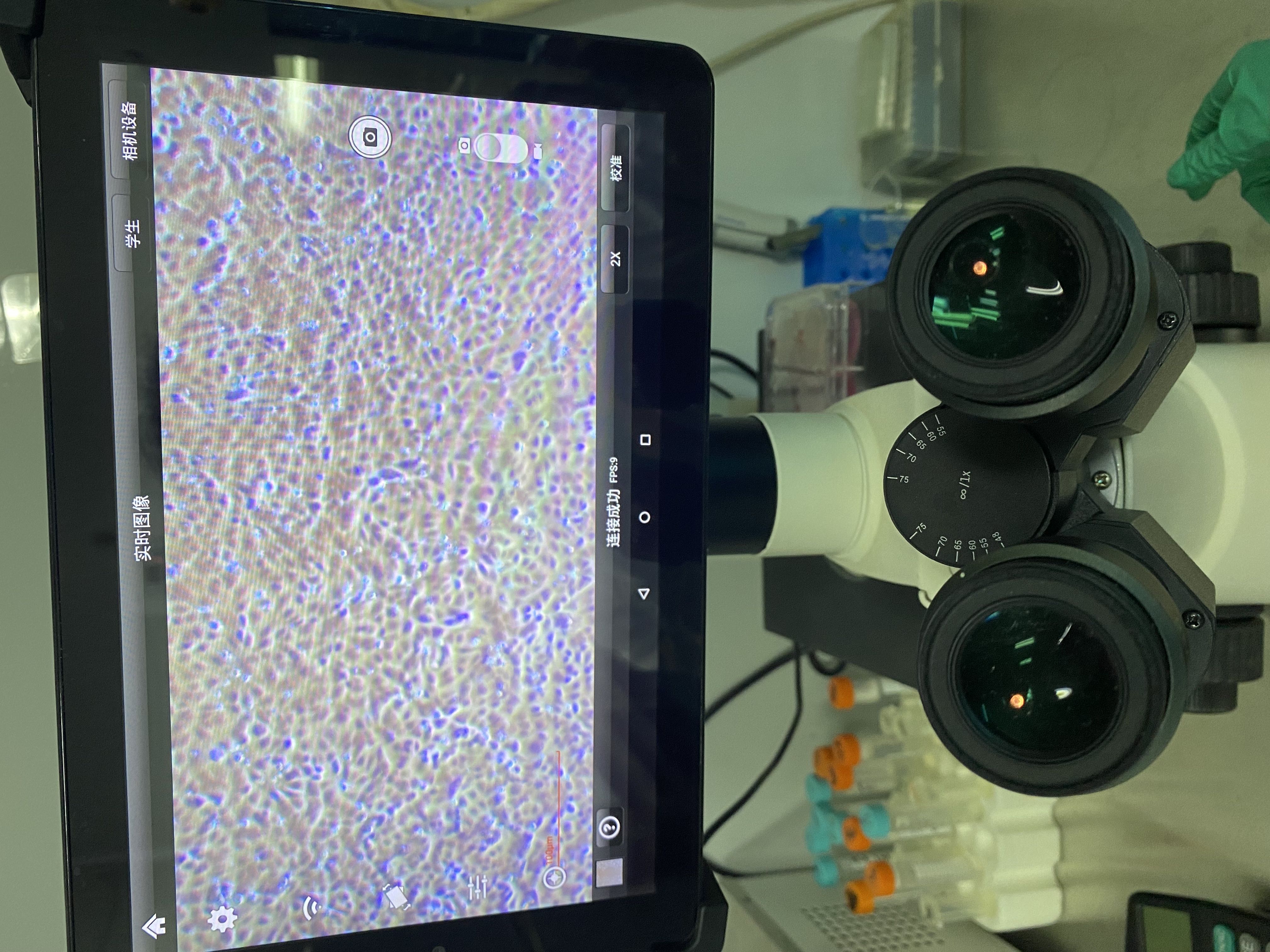

Supplement: Supplementary file 1 [file Data_Sheet_1.zip › supply materials/apoptosis cell photos/FS.jpg]

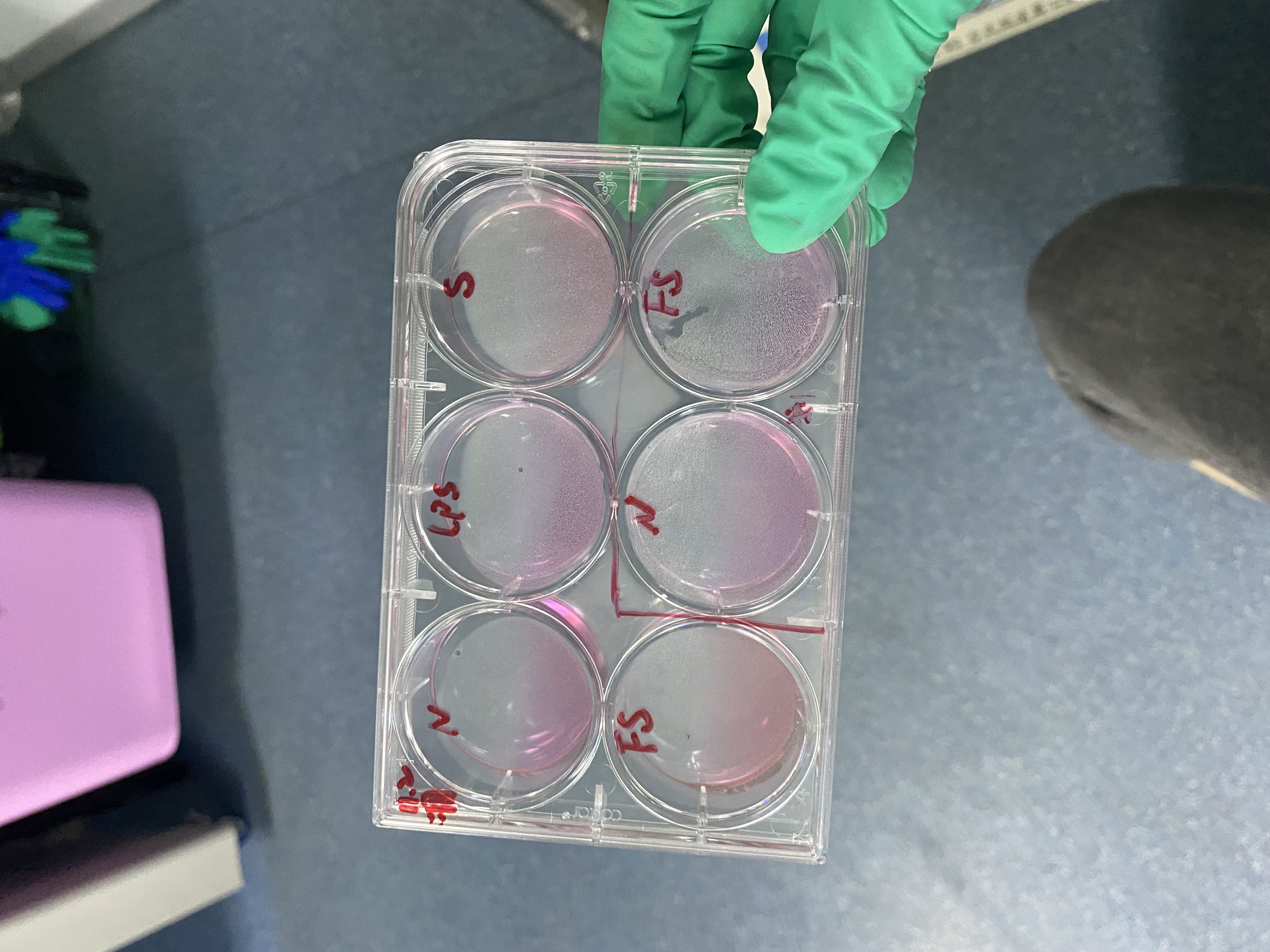

Supplement: Supplementary file 1 [file Data_Sheet_1.zip › supply materials/apoptosis cell photos/group.jpg]

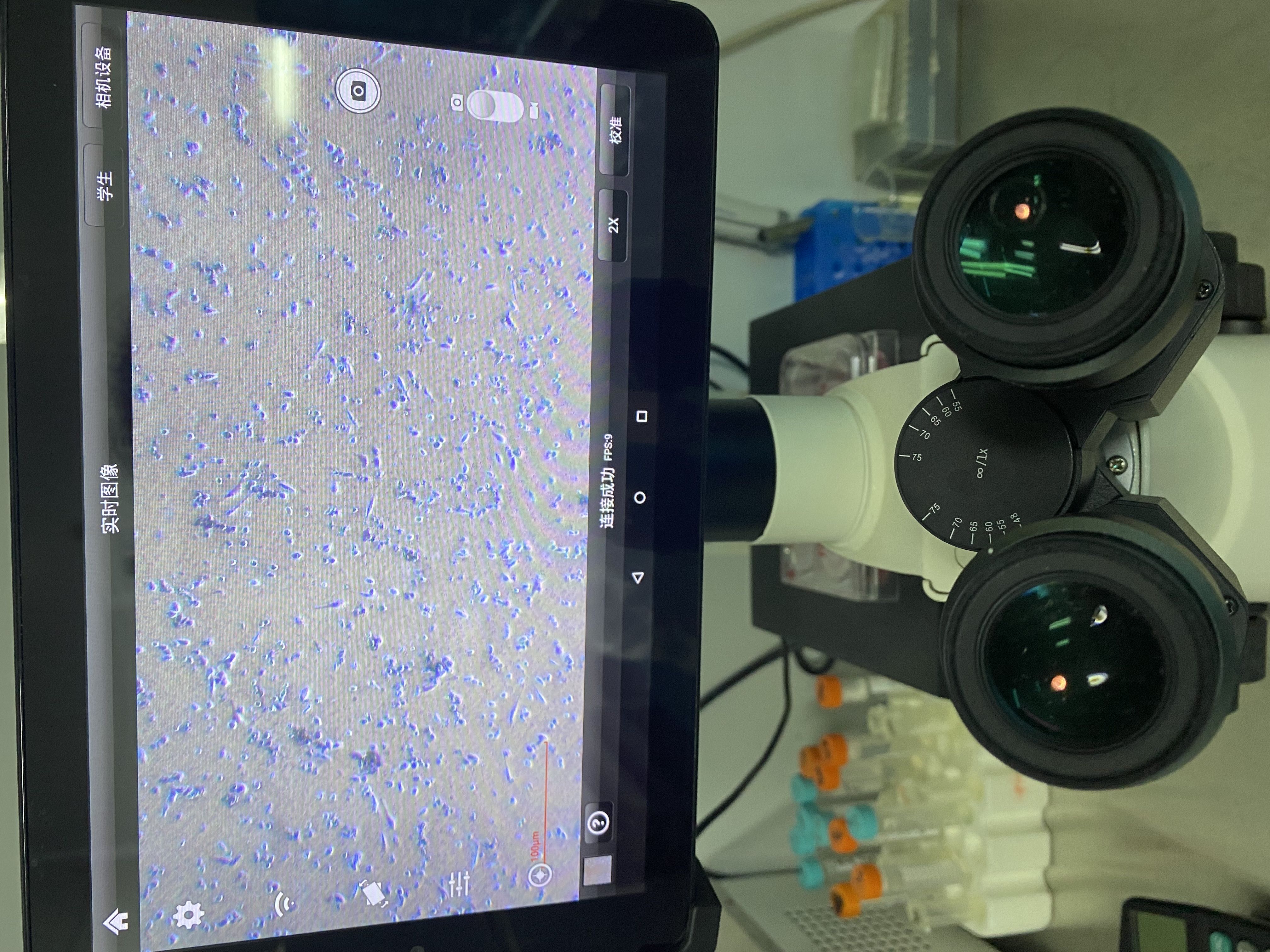

Supplement: Supplementary file 1 [file Data_Sheet_1.zip › supply materials/apoptosis cell photos/M.jpg]

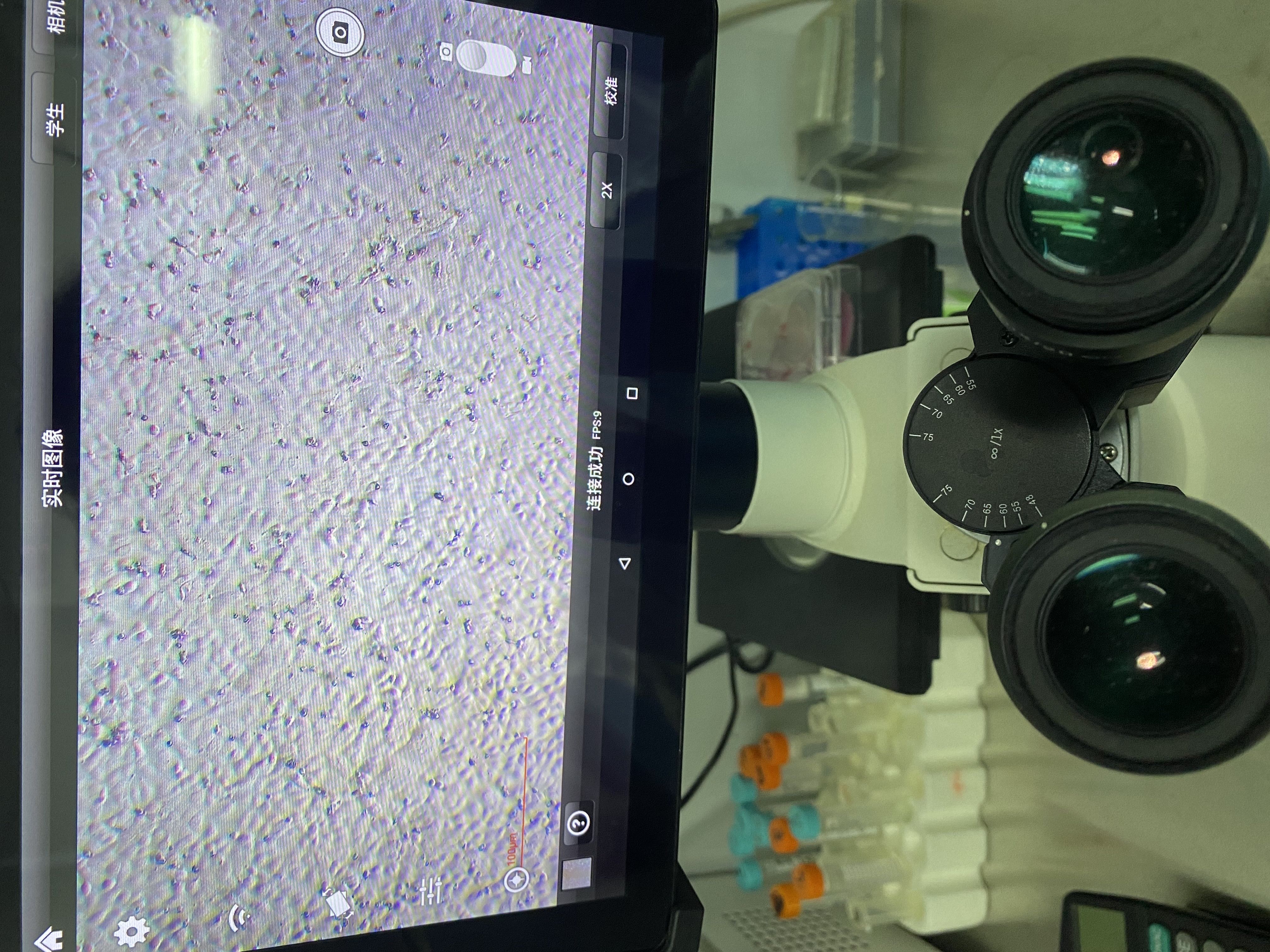

Supplement: Supplementary file 1 [file Data_Sheet_1.zip › supply materials/apoptosis cell photos/N.jpg]

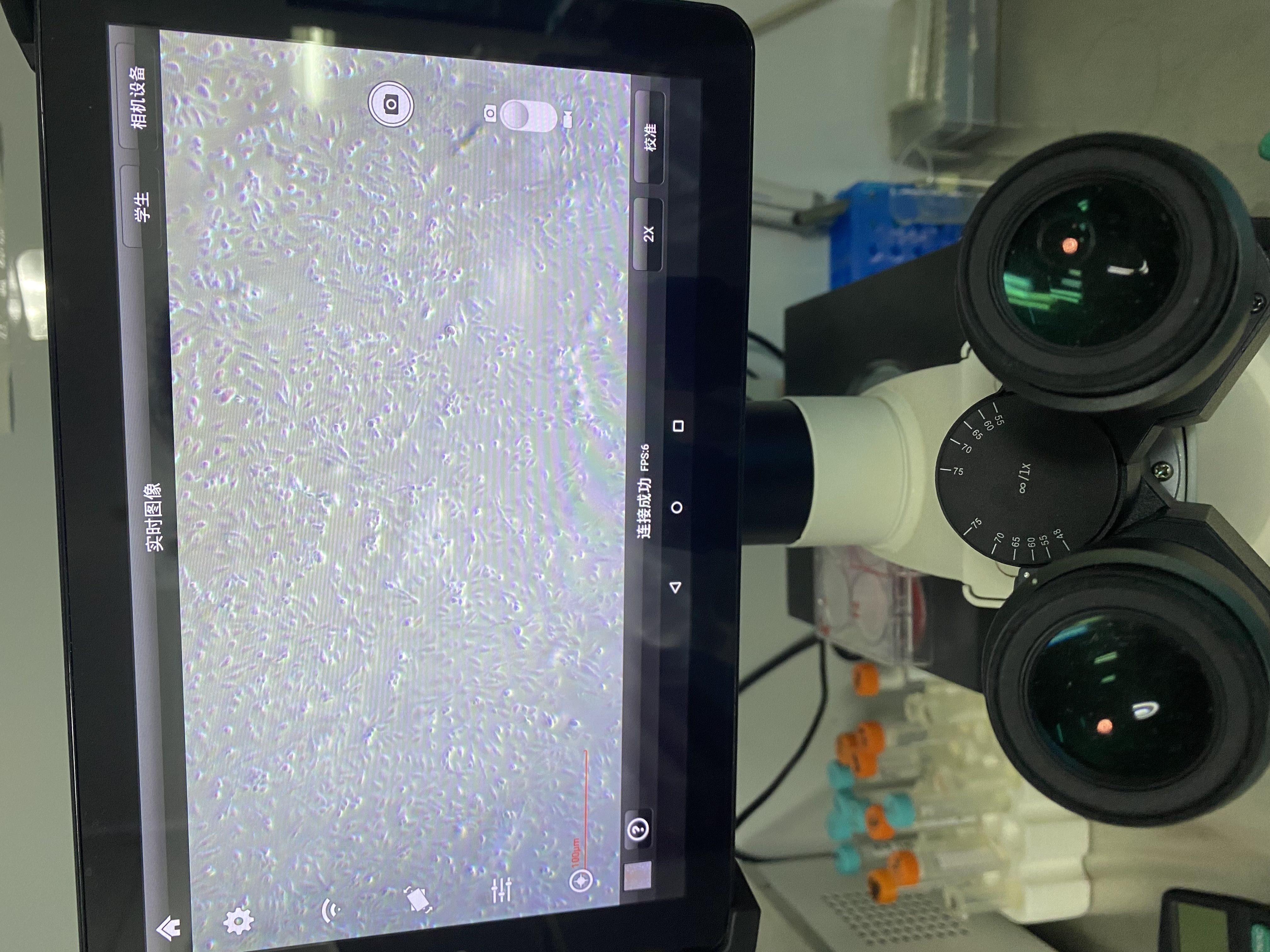

Supplement: Supplementary file 1 [file Data_Sheet_1.zip › supply materials/apoptosis cell photos/S.jpg]

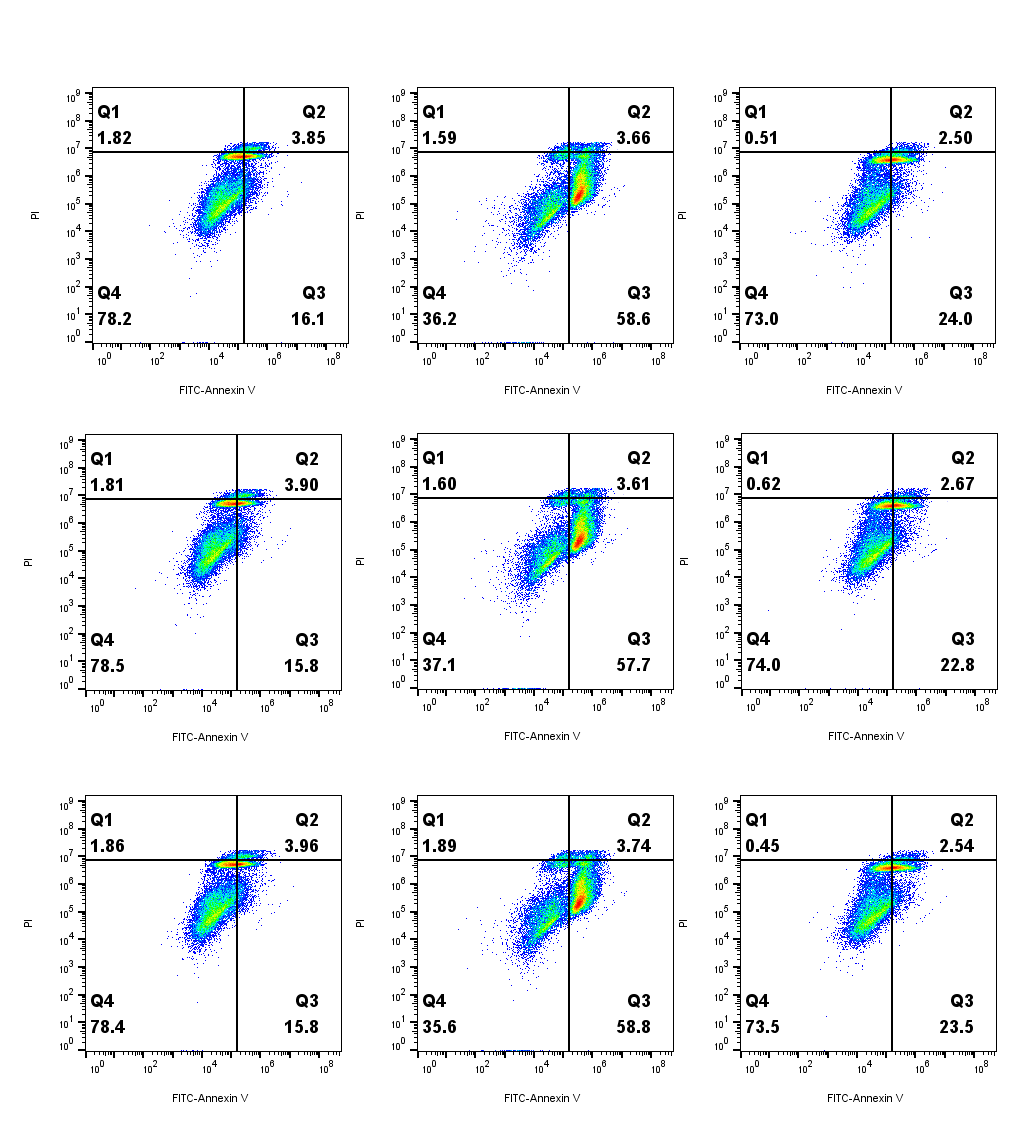

Supplement: Supplementary file 1 [file Data_Sheet_1.zip › supply materials/Flow cytometry-apoptosis.tiff]

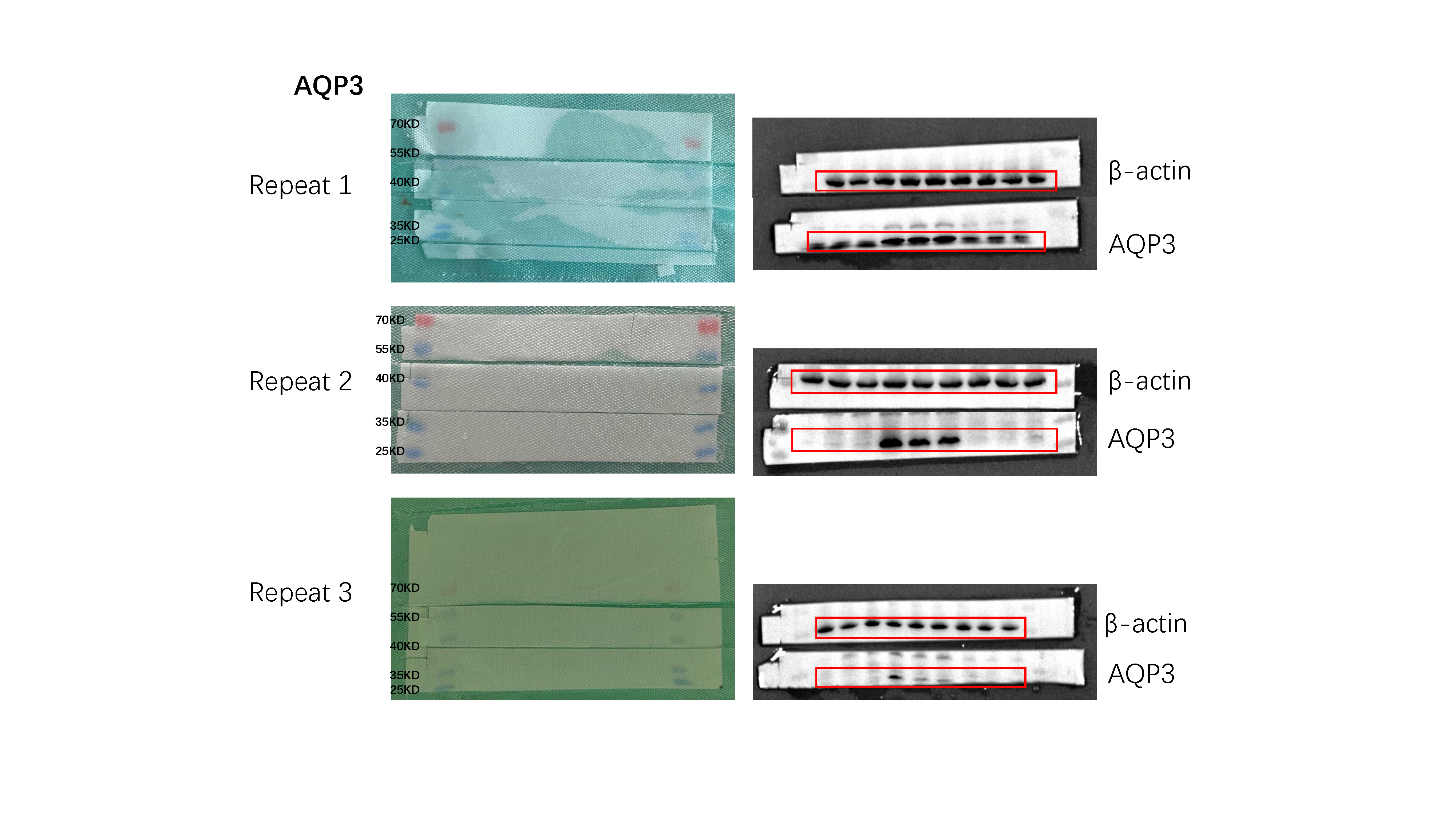

Supplement: Supplementary file 1 [file Data_Sheet_1.zip › supply materials/WB-AQP3.tiff]
